# Improving Osteoblast Response *In Vitro* by a Nanostructured Thin Film with Titanium Carbide and Titanium Oxides Clustered around Graphitic Carbon

**DOI:** 10.1371/journal.pone.0152566

**Published:** 2016-03-31

**Authors:** Giovanni Longo, Caterina Alexandra Ioannidu, Anna Scotto d’Abusco, Fabiana Superti, Carlo Misiano, Robertino Zanoni, Laura Politi, Luca Mazzola, Francesca Iosi, Francesco Mura, Roberto Scandurra

**Affiliations:** 1 Istituto di Struttura della Materia, CNR, Via del Fosso del Cavaliere 100, 00133, Roma, Italy; 2 Ecole Polytechnique Fédérale de Lausanne, SB IPSB LPMV, BSP 409 (Cubotron UNIL), R.te de la Sorge, CH-1015, Lausanne, Switzerland; 3 Dipartimento di Scienze Biochimiche, Università di Roma ‘La Sapienza’, Piazzale Aldo Moro 5, 00185, Roma, Italy; 4 Dipartimento di Tecnologie e Salute, Istituto Superiore di Sanità, Viale Regina Elena, 299, Roma, Italy; 5 Romana Film Sottili, Anzio, Roma; 6 Dipartimento di Chimica, Università di Roma ‘La Sapienza’, Piazzale Aldo Moro 5, 00185, Roma, Italy; Institute for Materials Science, GERMANY

## Abstract

**Introduction:**

Recently, we introduced a new deposition method, based on Ion Plating Plasma Assisted technology, to coat titanium implants with a thin but hard nanostructured layer composed of titanium carbide and titanium oxides, clustered around graphitic carbon. The nanostructured layer has a double effect: protects the bulk titanium against the harsh conditions of biological tissues and in the same time has a stimulating action on osteoblasts.

**Results:**

The aim of this work is to describe the biological effects of this layer on osteoblasts cultured *in vitro*. We demonstrate that the nanostructured layer causes an overexpression of many early genes correlated to proteins involved in bone turnover and an increase in the number of surface receptors for α3β1 integrin, talin, paxillin. Analyses at single-cell level, by scanning electron microscopy, atomic force microscopy, and single cell force spectroscopy, show how the proliferation, adhesion and spreading of cells cultured on coated titanium samples are higher than on uncoated titanium ones. Finally, the chemistry of the layer induces a better formation of blood clots and a higher number of adhered platelets, compared to the uncoated cases, and these are useful features to improve the speed of implant osseointegration.

**Conclusion:**

In summary, the nanostructured TiC film, due to its physical and chemical properties, can be used to protect the implants and to improve their acceptance by the bone.

## Introduction

The low density, strong robustness and low cost, coupled with high biocompatibility, make titanium the material of choice for most orthopaedic and dental implants. Many studies have evidenced that various factors, as chemistry, wettability, roughness or substrate microstructural features, may contribute to the biocompatibility of titanium or of modified titanium surfaces. Among these factors the roughness of the surface is retained of great importance: for this reason titanium surfaces of implants are mechanically treated or acid etched in order to bestow the opportune micro and macro roughness values which are beneficial to an efficient osseointegration and implant duration [1,2]. Furthermore, due to the well-known high affinity of titanium for oxygen [3,4] the surfaces of these implants are naturally passivated by a layer of titanium oxides, mainly TiO_2_ [5,6], which is considered the origin of their excellent corrosion resistance and high biocompatibility[7,8]. However, in the harsh conditions of the biological environment, this passivating layer tends to grow, to form a consistent non-metal layer at the interface between tissue and implant, which could become loosely attached to the bone and may be fractured exposing the bulk titanium to the attack of the biological environment. As consequence, titanium and TiO_2_ nanoparticles could be freed from bulk titanium implant, flooding the body [9] and, due to their size, being easily taken up by cells, inducing toxic reactions.[10,11]

Furthermore, the chemistry of these non-metallic titanium layers could stimulate the cells to produce a large number of small adhesion points which induce fibrinogenesis and lead to soft tissue encapsulation of implants, micromotion, implant loosening and, finally, to implant failure.[12–14] This outcome is not uncommon, can be found in a non-negligible portion of implants, and has a very large consequence in human distress and social costs.

To increase the clinical success of the implants and to decrease the time necessary for healing and integration of implants into the bone, many strategies have been adopted. Among the most successful are the modification of the implant surfaces by physical methods, the coating of the implants with layers of bioactive materials or the functionalization of the implant surfaces with biomimetic molecules to give a major bioactivity to the implant.[15] This latter strategy has given some very interesting results, as in the use short peptide chains, which include sequences found in some adhesion molecules like fibronectin.[16] Such strategy is very efficient and follows a natural pathway; however, it presents some disadvantages due the high costs in the synthesis of peptides and in the manufacturing of the functionalization with the biomimetic peptides on the surface of titanium implants.

On the other hand, several studies have proposed the coating of the surface of the titanium implants with biocompatible layers. A typical example is the coating with hydroxyapatite, which bestows a major bioactivity to the implant. However, the composition of this layer is different from the titanium of the implant and has a different resistance to the harsh conditions of a living biological environment. This could result in fractures in the coating layer, which would allow biological fluids to reach the bulk titanium, generating titanium particles. The consequence of this could be inflammation, growth of a fibrotic tissue and loosening of the implant.[2,14]

Previously, we have shown how to coat titanium implants with a hard layer, whose chemical structure can exert a double effect, protecting the bulk titanium against the harsh conditions of biological environment and stimulating the adhesion and activity of osteoblasts. This could lead to improved osseointegration of the implant and to decreased healing time.[17] Recently we described the coating of the titanium surface with a very hard and highly biocompatible layer containing titanium carbide (TiC), since it is a combination of two biocompatible materials as carbon and titanium. We used an Ion Plating Plasma Assisted (IPPA) deposition, which allows treating many samples per cycle and opens the way to its use in industrial applications. The resulting layer protects the bulk titanium of the implant and has a chemical composition, as characterized with X-ray Photoelectron Spectroscopy (XPS), that comprises a mixture of titanium carbide (36%) and titanium oxides (64%).[18,19] The preliminary biological results showed that this layer stimulated the growth of osteoblasts *in vitro*, better than the uncoated titanium. Furthermore, it retained the nano and micro sized roughness of the underlying substrate, which are well known to be of great importance for a good implant integration.[20–22] These pioneering results encouraged new experiments to understand if the conditions used in the preliminary deposition by the IPPA apparatus were susceptible to improvement. The latter had been obtained by the innovative use of a magnetron-activated TiC as the source of both Ti and C, which produced a nanostructured thin layer in which graphitic carbon was clustered with titanium carbide and titanium oxides. This optimization resulted in the deposition of a hard nanostructure with a thickness of about 500 nm, composed of 60% graphitic carbon, 15% titanium carbide and 25% titanium.[23] This is a novel application of graphitic carbon, a most versatile material which finds a wide application ranging from materials sciences, aerographite is considered the lightest structure in the world[24], to biology and nanomedicine. Indeed, there are abundant biomedical and nanotechnological applications of graphene[25] such as carbon nanotubes[26] which, due to their antimicrobial activity, high conductivity, optical transparency and surface-sensitive properties, are becoming increasingly studied.

In the present paper, we report that this optimized nanostructured TiC layer improves adhesion, proliferation and gene expression of Saos-2 osteoblasts and of human primary osteoblast cell cultures (hOB) compared with the results obtained on uncoated titanium.

## Materials and Methods

### Materials

Triton X-100, McCoy’s medium, Fetal Bovine Serum (FBS), phosphate-buffer saline (PBS), paraformaldeheide, fibronectin, methylene iodide, formamide, sodium cacodilate, collagenase type IV and the MTT (3-(4,5-dimrthylthiazol-2-yl)-2,5-di-phenyl tetrazolium bromide)-based colorimetric assay were obtained from Sigma-Aldrich (St. Louis, MO, USA). Penicillin/streptomycin, L-glutamine and trypsin were obtained from HyClone (HyClone, Logan, UT, USA). Transforming Growth Factor-β1 (TGF-β1) was acquired from PeproTech House, London, UK. α3β1 mouse monoclonal antibody-MAB-10624 and phalloidin Alexa Fluor 488-coniugated-PP-10052 were acquired from Immunological Sciences (Rome, Italy). Trizol reagent, DAPI, Alexa Fluor 568 goat anti-rabbit and Alexa Fluor 568 goat anti-mouse were bought from Invitrogen (Carlsbad, CA, USA). Anti-paxillin rabbit polyclonal antibodies (NBP1-19833) and anti-talin mouse monoclonal antibodies (NBP1-21642) were obtained from Novus Biologicals (Littlenton, CO, USA). Anti-tubulin monoclonal antibodies were purchased from Millipore (Billerica, Massachusetts, USA). The SYBR green PCR Master mix was acquired from Applied Biosystems and the TrypLe Select detaching buffer from Gibco (Thermo Fisher Scientific, Waltham, Massachusetts, USA). The TGF-β1 EIA kit ADA-900-155 was purchased from Enzo Life Sciences (NY, USA). The ALP assay colorimetric kit (ab83369) was obtained from Abcam (Cambridge, UK).

### Substrate preparation

The titanium samples used in this work were obtained using Titanium grade 2 (Ti = 99.85%; C = 0.0006%; Ni = 0.01%; O = 0.1%; H = 0.003%; Se = 0.03%) rods (Cerac, WI, USA) that were cut into disks (13 mm diameter, 2 mm thick) or tablets (30 x 30 mm, 2 mm thick. All the disks were blasted with 120 μm zirconia microspheres, which gave an averaged roughness of about 50 nm, uniformly distributed on the entire surface.

Upon preparation, half of the disks were coated with a TiC layer using an optimized Ion Plating Plasma Assisted deposition[17–19] described in detail elsewhere[23] to obtain a uniform and well-attached coating. In previous works, we performed an in-depth characterization of the morphological, microstructural chemical and mechanical properties of the disks. We characterized and compared the bare titanium disks and the nanostructured layer using several techniques. These analyses are recounted in our previous works and include Atomic Force Microscopy (AFM), Focused Ion Beam (FIB)/Scanning Electron Microscope (SEM), Transmission Electron Microscopy (TEM) and X-ray Photoelectron Microscopy (XPS) studies.[23] In particular, we demonstrated that the deposition of the film introduces only very little roughness variations, indicating that any modification of the biological response can be attributed solely to the different chemical properties of the substrate.[18]

All the substrates were thoroughly cleaned by immersion for 30 minutes in a 0.1% solution of Triton X-100, sonicated and rinsed with ultrapure water. Next, they were washed three times with hexane and air-dried. The samples, upon transfer for the biological tests, were also sterilized by autoclaving.

### Ethics disclosure

We obtained blood samples from consenting donors and bone fragments from consenting donors, who underwent surgical treatment, following the ethical procedures defined by the ethical committee of the Rome University La Sapienza. The donors consented in writing to the use of their samples, that the results would be completely anonymous and that the samples would not be used to obtain any relevant medical information on the donor. In view of this and of the lack of personal information that could be extracted from the use of the biological samples, the ethics committee of the Rome University La Sapienza approved this particular study.

### Cell cultures

The Saos-2 (HTB-85) cell line, a commercially available clonal human osteosarcoma cell line, was obtained from American Type Culture Collection (ATCC, Rockville, MD, USA); these cells are in early differentiation stage and can easily be used to study the effects on the proliferation and differentiation of human osteoblasts. Human primary osteoblasts (hOB) were isolated from bone fragments, of at least five different patients, three male and two female healthy patients of 50–70 years old submitted to surgical treatment. The bone fragments were washed in sterile phosphate-buffer saline (PBS), minced and treated with 1mg/ml collagenase type IV and 0.25% trypsin for 1 hour at 37°C with gentle agitation. The procedure was repeated one more time; cells from the first and second digestion were collected by centrifugation. In order to avoid the variability among subjects, cells from the five patients were pooled, divided in lots and stored at -20°C until used.

Both Saos-2 and hOB (from 2.5 to 18 x10^4^ cells) were seeded on test substrates (untreated and treated titanium disks 13x2 mm or tablets 30x30x2 mm, respectively) at a density of 2x10^4^/cm^2^ and cultured for the requested times at 37°C in 5% CO_2_. Saos-2 and hOB were grown to 80% confluence in McCoy’s medium supplemented with L-glutamine, penicillin/streptomycin and 15% FBS. All the experiments were performed in McCoy’s containing 1% FBS.

### Contact angle and surface free energy

The wettability of the sample surface, before and after the TiC coating deposition, was investigated using a contact angle meter, developed in the laboratory of the Surface Engineering (Industrial and Mechanical Engineering Department—Roma Tre University). For these measurements, we used methylene iodide and two polar liquids (formamide and water).

We deposited 3 μl of each liquid on the TiC and on the uncoated samples. The contact angle measurements were performed with a custom device (Mazzola), at room temperature and humidity (22°C and 30% respectively).

Once the liquids were placed on the substrates, the resulting sessile drop was imaged for analysis. These images were “binarized” (using black and white colours to maximize the contrast) with the Analysis software (produced by Olympus Soft Imaging System) to improve the accuracy and the reliability of the contact angle measurements.

The surface free energy was calculated according to the Van Oss–Chaudhury—Good method, which represents the best, and innovative method for calculating the surface free energy of the materials.[27]

### Assessment of cell viability

To detect potential cytotoxic effects of the nanostructured TiC film, the survival of cells grown on uncoated and TiC coated titanium disks was evaluated using MTT (3-(4,5-dimrthylthiazol-2-yl)-2,5-di-phenyl tetrazolium bromide)-based colorimetric assay in accordance with manufacturer’s instructions. Briefly 1.5 x 10^5^ hOB cells in 500 μl McCoy medium were seeded on uncoated and TiC coated titanium tablets (30x30 mm) inserted into Petri-dishes and incubated at 37°C, with 5% CO_2_ for 3 and 7 days. At the end of incubation time 50 μl of MTT solution was added to the 500 μL of McCoy medium on each tablet and incubated for an additional 4 hours. Afterwards, the culture medium was gently removed from the tablets, then 150 μl of solvent (HCL 0.1N in anhydrous isopropanol) was added to each tablet to dissolve the MTT formazan crystals. The solutions were transferred into a 96 multiwall plate and spectrophotometric absorbance was measured at 570nm. The background at 690 nm was subtracted.

### ALP and TGFβ1 determination

After culturing the cells on Ti and on TiC, for the time required for the different experimental procedures, we lysed the cells and collected the cell supernatants. We measured the concentration (pg/ml) of Transforming Growth Factor-β1 (TGF-β1) in the cell supernatants by using TGF-β1 EIA kit (ADI-900-155).

The Alkaline Phosphatase (ALP) activity (U/ml) was measured in cell lysates using ALP assay colorimetric (kit ab83369). The experiments were conducted according to manufacturers’ instructions

### RNA extraction

After incubation on the chosen substrates, the cells were washed with cold PBS, scraped and the total RNA was extracted using 1ml of Trizol reagent in accordance to the manufacturer’s instruction. RNA was stored at -20°C until used. cDNA was synthesized from 1 μg of total RNA, using reverse transcriptase Improm II (Promega Corporation, Madison, WI, USA) in accordance with the manufacturer’s instructions, and analysed by Q-RT-PCR.

### Quantitative-Real-time Polymerase Chain Reaction (Q-RT-PCR)

Q-RT-PCR analysis was performed using an AB Prism 7300 (Applied Biosystems, Foster City, CA, USA). The primers were designed using Primer Express software (Applied Biosystems) and were synthesized by Primm (Milano, Italy). The results were analysed by using the Sequence Detection Systems software (Applied Biosystems), which automatically recorded the threshold cycle (C_t_). Untreated cell sample (CTL) was used as calibrator; the fold change for CTL was 1.0. Target gene C_t_ values were normalized against GAPDH.

**Fig 1:**
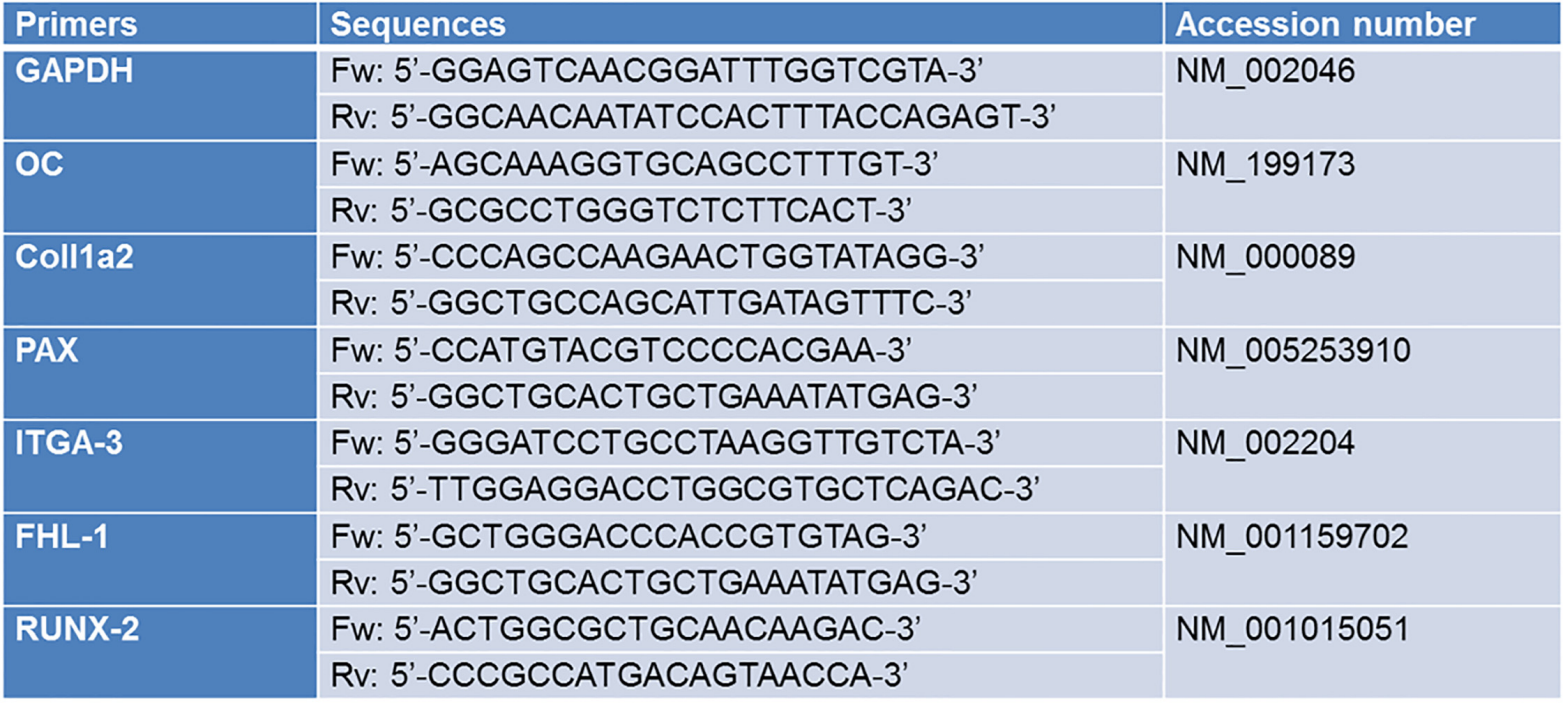
Primer sequences used in Q-RT-PCR.

The amplification was carried out with 50 ng of cDNA, in 96-well plates, using SYBR green PCR Master mix in 20 μl volume. Each sample was analysed in triplicate. PCR conditions were: 95°C for 10 min followed by 40 cycles of 95°C for 15 s and 60°C for 1 min. The primer sequences are reported in Fig 1. The data were analysed using the 2^-ΔΔCt^ method [28] and are expressed as fold change compared to mRNA extracted from cells cultured on Ti disks.

### Immunofluorescence

To analyse the cells by fluorescence microscopy, to detect differences in cell growth on Titanium (Ti) and on the nanostructured Titanium carbide (TiC) film, we coated by IPPA (in optimal deposition conditions) some glass slides with very thin films (10.5 nm) of Ti or TiC. The layers allow the transmission of 25% of the light, which was sufficient to perform fluorescence microscopy analyses. We used XPS characterization to determine the chemical composition of the layers of titanium and TiC on the glass slides used for immunofluorescence experiments and to determine that they had the same composition with the corresponding titanium disks. This analysis is reported in Fig A in S1 File and demonstrates that the corresponding substrates had identical composition.

Due to these results, we were able to perform immunofluorescence analyses directly on the treated glass slides and to compare these results with those obtained from cells grown on the disks.

To quantify the surface receptors, we spread 2 x 10^4^ cells, both Saos-2 and hOB, in 500 μl McCoy medium volume, on Ti or TiC coated glass slides and left to grow for 96 h at 37°C, 5% CO_2_. After washing with PBS, we blocked the cells with 5% bovine serum albumin and incubated them overnight at 4°C with anti-integrin α3β1 mouse monoclonal antibody, with anti-paxillin rabbit polyclonal antibody with anti-talin mouse monoclonal antibody and with anti-tubulin monoclonal antibody. All primary antibodies were used at 10 μg /ml. After incubation, we washed the cells with PBS and incubated them for 1 hour at room temperature with the opportune Alexa Fluor secondary antibody, Alexa Fluor 568 goat anti-rabbit and Alexa Fluor 568 goat anti-mouse, both diluted 1:500. Actin was detected by incubating the cells with phalloidin Alexa Fluor 488-coniugated.

To detect the nuclei, we washed the slides and we incubated them with DAPI. All the images show representative images collected from three independent experiments. In all cases, the images were collected at 63x or 100x magnification using a Leica DMI 6000B fluorescent microscope (Leica Microsystems CMS GmbH Wetzlar, DE) equipped with a Leica AF6000 System.

### Optical Microscopy

The optical images were obtained using a commercial inverted optical microscope (Axio Observer.A1, Carl Zeiss, Göttingen, DE) and imaging the cells growing on coated glass slides. In all images, we used a 40x objective and the images were acquired with a built-in camera. The cells were imaged in the growing medium after a 6h incubation at 37°C, 5% CO_2_. To ensure the statistical value of the images, we investigated minimally 100 cells per sample and we studied three different preparations of each glass substrate.

### Atomic Force Microscopy

We seeded 2.5 x10^4^ Saos-2 cells on treated and untreated titanium disks (13x2mm), we fitted each substrate into a 10 cm Petri-dish and we cultured them for 6h in McCoy medium at 37°C, 5% CO_2_. After this time-period we fixed the cells with 0.5% glutaraldehyde in 10mM PBS (pH 7.4) overnight at 37°C, inserted them in a custom analysis chamber, which was flushed with sterile PBS and proceeded immediately to perform AFM imaging.[29]

The AFM imaging was performed using a Nanowizard III (JPK Instruments AG, Berlin, DE). We performed the analyses using Bruker DNP-10 cantilevers (Bruker probes, Berlin, DE), choosing the ones with a nominal spring constant of 0.06 N/m. Before each experiment, we calibrated the mechanical properties of the tip using the JPK software. All the presented images were obtained by working in the quantitative imaging (QI) modality, an evolution of the force-volume mode in which the AFM tip is placed in fast oscillation over the sample and the deformation of the cantilever is recorded to reconstruct an image formed by a large number of force distance (FD) curves. Typical images contain up to 256x256 pixels and, for every pixel, 2048 points per FD curves were collected. The length of the curves was 1 μm and the imaging speed ranged from 0.1 to 3 lines per second. The tip-sample interaction was limited to a maximum cantilever deflection of 40 nm (~ 2.5 nN). The data files were recorded on at least 30 cells per condition and the measurements were performed on three independently prepared substrates.

Processing was done in a semi-automated way with the JPK data processing software and the images were flattened.

### Scanning Electron Microscopy

The cell morphology, adhesion, and spreading patterns were investigated by scanning electron microscopy (SEM). hOB and Saos-2 cells (1 x10^4^ cells) were seeded on three sets of five treated and untreated titanium disks (13x2mm): each set was fitted into a 10 cm plastic dish and cultured in McCoy medium at 37°C in 5% CO_2_.

After 6 or 24 h incubation, the cells were fixed with 2.5% glutaraldehyde in 0.1 M sodium cacodylate buffer (pH 7.4) overnight at 4°C and postfixed with 1% OsO_4_ in 0.1 M sodium cacodylate buffer for 1 h at room temperature. The samples were then dehydrated through a graded series of ethanol solutions, critical point dried and gold sputtered, and examined with a QuantaTM 650 FEG electron microscope (Fei, Eindhoven, The Netherlands). The SEM micrographs were taken with a typical voltage of 10 kV and spot size of 3 (arbitrary units).

### Detaching test

The Saos-2 cells (5x10^5^) in 500 μl McCoy medium were seeded on three sets of three uncoated and TiC coated tablets (30x30 mm): each set was fitted into a 10 cm plastic dish, cultured at 37°C in 5% CO_2_ for six hours. At the end of incubation time, the culture medium was gently removed from the tablets, adherent cells were rinsed in 10 mM PBS pH 7.4 and covered for 10 minutes with 500 μl of the TrypLE Select CTS detaching buffer, which contains a recombinant microbial protease. After 10 minutes the liquid was removed and dislodged cells were counted in a Thoma-Zeiss counting chamber under the optical microscope.

### Single cell force spectroscopy: measurement of the adhesion forces

To measure in a direct and quantitative way the interaction between the cells and the differently treated substrates, we performed some adhesion experiments measuring directly, using an AFM cantilever, the interactions forming between a cell and the substrate. The AFM modality we used is called single cell force spectroscopy (SCFS) and consists in using a single cell as AFM tip to probe its interactions with a surface.[30–32].

These experiments were carried out using a JPK Nanowizard III (JPK, Berlin, DE) microscope and using DNP-10 cantilevers (Bruker, USA). We functionalized the cantilevers with fibronectin (10 mg/ml for 15 minutes). The sensor was then inserted in the analysis chamber, which was flushed with a cellular nourishing buffer containing a small concentration of live cells.[29] At this point, we used the AFM coarse and fine movement capabilities to “fish” a single cell, attaching it near the apical region of the cantilever. We left the cell to grow on the cantilever for approximately 30 minutes and then we used this modified tip for the SCFS analyses. The cantilever bearing the living osteoblast on its apical area was, then transferred to the glass, titanium or TiC coated substrates to perform the SCFS experiments. We approached the cell to each substrate with a constant speed of 1 μm/s until the osteoblast was in contact with the surface. Next, we maintained a constant cell-substrate interaction (maximum applied force 6nN) for some seconds (5, 20 and 60 seconds were probed) and finally retracted.

We exploited the high force resolution of the cantilever sensor to ensure that the cell-substrate interaction was kept constant and with an overall force that did not cause stress to the cell. After predetermined times, we pulled away the cantilever, and measured the force curves arising from the osteoblast detaching from the different substrates. In all cases, at the end of each curve, the cell was still firmly attached to the apical area of the cantilever, as confirmed by the optical images we collected while the experiments were carried out.

The area difference between each corresponding approach and retraction curve was used to define the adhesion value for each experiment. The typical length of these force curves was 10 microns and the Zeiss conventional optical microscope coupled with the JPK microscope was used to control the cell’s condition and its continued presence on the cantilever. To obtain a good statistical significance, we performed minimally 100 curves per cell per substrate and we repeated the experiments using 10 different cells.

### Blood clotting

For the qualitative analysis of the blood clot formation, uncoated and TiC coated titanium disks were put into a 24-well dish (NuncA/S, Roskilde, Denmark) sterilized by exposing to UV for 2h, then exposed to human Whole Blood (hWB) or human Platelet-Rich Plasma (hPRP).

In the first case, the wells were filled with 3 ml of freshly drawn hWB (obtained from consenting donors) and incubated 4 min at RT. The excess liquid was then discarded and the discs were rinsed three times in a saline solution.

In the second case, the sterilized substrates were incubated with 3 ml of hPRP at 37°C, 5% CO_2_ for 90 min. After the incubation time, the disks were set free of liquids by suction and rinsed in a saline solution.

After the preparation, all disks were fixed in 2.5% glutaraldehyde in 0.1M cacodilate buffer pH 7.4 for 1 h at 4°C, dehydrated through a graded series of ethanol solutions and air dried. The samples were then sputtered with 10 nm of chromium with a Quorum Q150T Sputter Coater (Quorum Technologies, Laughton, UK) and examined with a FESEM (Field Emission Scanning Electron Microscope, Auriga Zeiss).

### Statistical analysis

Each experiment was repeated using five independent cell samples and each sample was analysed in triplicate. The statistical significance of the differences between mean values was determined by a two-tailed t test; p value of not more than 0.05 was considered significant. Where appropriate, results are expressed as the mean ± standard error of the mean (SEM).

## Results

### Wettability and surface free energy

The biocompatibility of the biomedical prostheses is due not only to the affinity of its chemistry and morphology with the host tissues, but also to some physical parameters such as the wettability, defined as the contact angle formed by a water drop.[27,33–41] To achieve a good adhesion to a substrate, firstly of the proteins of the extracellular fluid (i.e. fibrinogen) and then of the overall cells, it has been demonstrated that the wettability of the substrate should have a value of about 70°. Furthermore, the adsorption appears to be improved on hydrophobic surfaces, compared to hydrophilic ones. We show in Table 1 how the water contact angle of TiC coated samples is 70.5°± 2.3, while uncoated substrates have a wettability angle of 60.0°±2.1.

**Table 1:** Contact angle on uncoated and TiC coated titanium disks.

	Water [°]	Methylene iodide [°]	Formamide [°]
**Uncoated sample**	60.0 ± 2.1	30.9 ± 1.8	32.8 ± 0.7
**TiC coated sample**	70.5 ± 2.3	52.0 ± 0.2	28.8 ± 0.6

Since the cell membrane is polarized and interacts directly with the surface, it is extremely important to study and control the surface free energy of the substrates. Our measurements, show that the surface free energy of a TiC coated sample is smaller than the uncoated substrate but, as shown in Table 2, this reduction leaves the TiC coated sample with almost only the basic fraction of the surface free energy (γ-). This physical and chemical analysis indicates that the TiC layer will be well accepted by the biological tissues and is predictive of a good cellular adhesion.

**Table 2:** Surface free energy parameters for uncoated and TiC coated titanium disks.

	γ_tot_ [mJ/m^2^]	γ_d_ [mJ/m^2^]	γ_p_ [mJ/m^2^]	γ^+^ [mJ/m^2^]	γ^-^ [mJ/m^2^]
**Uncoated sample**	51.54	43.02	8.52	1.44	12.56
**TiC coated sample**	44.83	44.7	0.13	0	11.94

γ_tot_ = surface total free energy; γ_d_ = dispersed component; γ_p_ = polar component; γ^+^ = acid fraction; γ^-^ = basic fraction.

### Toxicity assays

To establish that this optimized nanostructured TiC layer was devoid of any toxicity and negative effects on the viability of the cells, we cultured hOB cells on uncoated and TiC coated titanium disks. We incubated the cells for 3 and for 7 days and we measured the cell proliferation through the MTT test, which may then be at a same time a toxicity and a vitality test.

**Fig 2:**
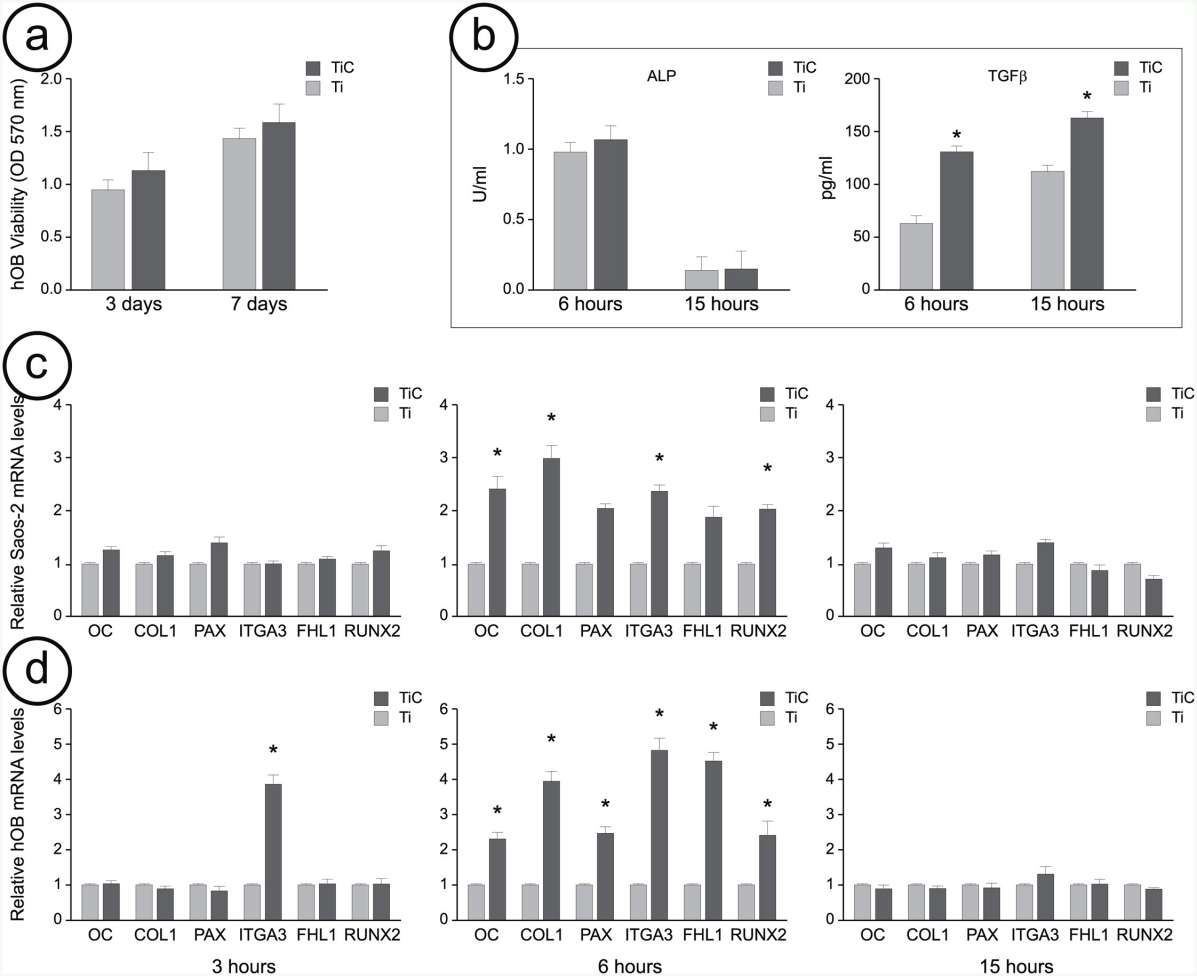
Viability, ALP and TGFβ1 production and gene expression of osteoblast cells cultured on uncoated and TiC coated titanium disks. **Panel a:** Cellular viability, assessed by MTT (3-[4,5-dimethylthiazol-2-yl]-2,5-di-phenyltetrazolium bromide) method. The human primary osteoblasts (hOB) (2 x 10^4^) were cultured for 3 and 7 days on uncoated titanium (Ti) disks (light grey bars) and on disk coated with nanostructured TiC layer (dark grey bars). Formazan extracted from cultures was determined spectrophotometrically at 570 nm. **Panel b:** ALP activity was quantitatively determined by colorimetric assay into cell lysate and TGFβ1 was quantitatively determined by ELISA into culture medium obtained from hOB cultured on uncoated Ti and on TiC coated disks. **Panel c**: Gene expression of osteocalcin (OC), collagen1a2 (COL1), paxillin (PAX), integrin α3β1 (ITGA-3), Four and Half LIM domains protein (FHL1) and Runt related transcription factor 2 (RUNX-2) obtained from the mRNA extracted from Saos-2 cells cultured 3, 6 and 15 hours on uncoated Ti and on TiC coated disks and analyzed by Q-RT-PCR. **Panel d**: Same analysis on mRNA obtained from hOB. All the presented results are reported as relative mRNA levels and expressed as fold change compared to mRNA extracted from cells cultured on uncoated titanium disks. Results represent the mean +/- Standard Error of the mean of data obtained by five independent experiments, performed using five cell samples, and each sample was analyzed in triplicate with a standard deviation among triplicate ranging between 0,38 and 0,04. * Indicates P-value < 0.05.

As shown in Fig 2a, after 3 days the proliferation of the cells grown on the TiC coated titanium disks was about 20% higher than that of the osteoblasts grown on the uncoated titanium disks, while after 7 days this value was 10%.

We also compared their ability to produce osteogenic differentiation factors, such as Transforming Growth Factor β1 (TGFβ1) and Alkaline Phosphatase (ALP). We measured their production by hOB grown on both Ti and TiC surfaces for 6 and 15 hours. The results recounted in Fig 2b show how the cells that were incubated on TiC substrates produced similar amounts of ALP compared to those produced by cells cultured on Ti. The amount of ALP was lower after 15 hours of incubation compared to the 6 hours culture both for the cells grown on Ti and for those on TIC. On the other hand, after 6 hours, the production of TGFβ1 for the cells cultured on TiC was higher in a statistically significant way when compared to the cells grown on Ti. This difference was reduced after 15 hours culture, but it was still statistically significant. The viability coupled with the increase in the production of osteogenic differentiation factors, such as TGFβ, for the cells grown on the titanium disks coated with nanostructured TiC, demonstrate that the chemistry of the layer is devoid of any toxicity and that, on the contrary, it has a slight effect on cellular viability.

### Gene expression investigation

After these preliminary toxicity tests, we investigated the effect of the chemical composition of the TiC layer on the gene expression of proteins involved in bone turnover.

We explored, for Saos-2 cell lines and hOB, both the short-term (3 and 6 hours) and the long-term (15 hours) effects of the coating. We compared the effects of the substrate at 3h, 6h and 15h on the mRNA expression level of several genes involved in bone turnover and cell adhesion, such as osteocalcin (OC), collagen 1a2 (COL1), paxillin (PAX), integrin α3β1 (ITGA-3), Four and Half LIM domains protein (FHL1) and Runt related transcription factor 2 (RUNX-2) (Fig 2c and 2d). After 3 hours of incubation on the two different substrates (light and dark grey bars), all genes resulted slightly up-regulated in Saos-2 cells, whereas the mRNA level of ITGA-3 gene was highly up-regulated in hOB. After 6 hours, we could evidence how TiC (dark grey bar) induced an up-regulation of the mRNA level of all the tested genes compared to cells seeded on Ti (light grey bar). This up-regulation was significant in the case of Saos-2 cells and even more evident for hOB. After the longer period of 15 hours of incubation, the increase in gene expression levels had disappeared for both Saos-2 and hOB.

### Investigation of surface receptors

To study the cross talk between the cell and the chemistry of the environment, we used the commercial microscopy glass slides coated with titanium or of nanostructured TiC. Since the chemical composition of these substrates was identical to those obtained on titanium disks (as reported in S1 File) we could perform immunofluorescence assays to quantify the surface receptors for paxillin, integrin and talin, as well as actin and tubulin, the components of the cellular cytoskeleton.[42–53]

**Fig 3:**
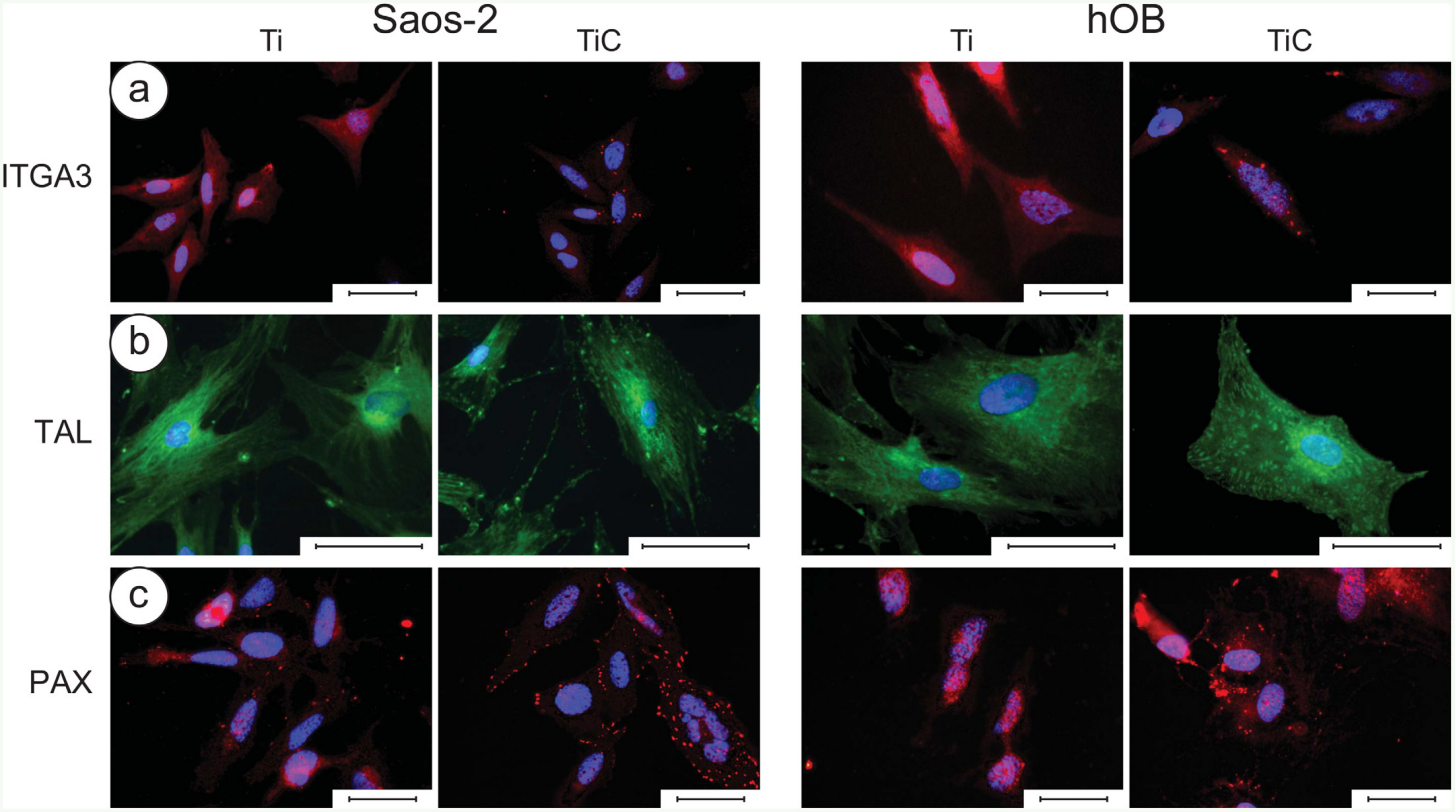
Immunofluorescence images of integrin α3β1, talin and paxillin in Saos-2 cells and in human primary osteoblasts (hOB). **Panel a:** The Saos-2 (left panels) and the hOB cells (right panels) were grown for 96 h on glass slides coated with 10.5 nm of titanium or the nanostructured TiC layer, treated with primary monoclonal antibodies against integrin **α3β1** (10 μg/ml). **Panel b:** The cells were treated with primary monoclonal antibodies against talin (10 μg/mL). **Panel c:** Cells were treated with primary monoclonal antibodies against paxillin (10 μg/ml). In all cases, the treatment was followed with Alexa Fluor 568 goat anti-mouse secondary antibodies, diluted 1:500 and the nuclei were stained with DAPI. The images were collected with a magnification of 63X for ITGA and PAX and of 100x for TAL, and the bar represents 100 μm.

Analysing the immunofluorescence images (Fig 3), we were able to highlight that the interaction with the chemistry of the nanostructured TiC layer produced, both on Saos-2 cells and hOB, a higher number of receptors of Integrin α3β1 (ITGA3) red spots, talin (TAL) green spots and paxillin (PAX) red spots, all members of Focal Adhesion plaques (Fig 3a, 3b and 3c respectively). This suggests that the TiC-treated glasses induced a stimulating effect on the cells when compared to the Ti coated glasses.

**Fig 4:**
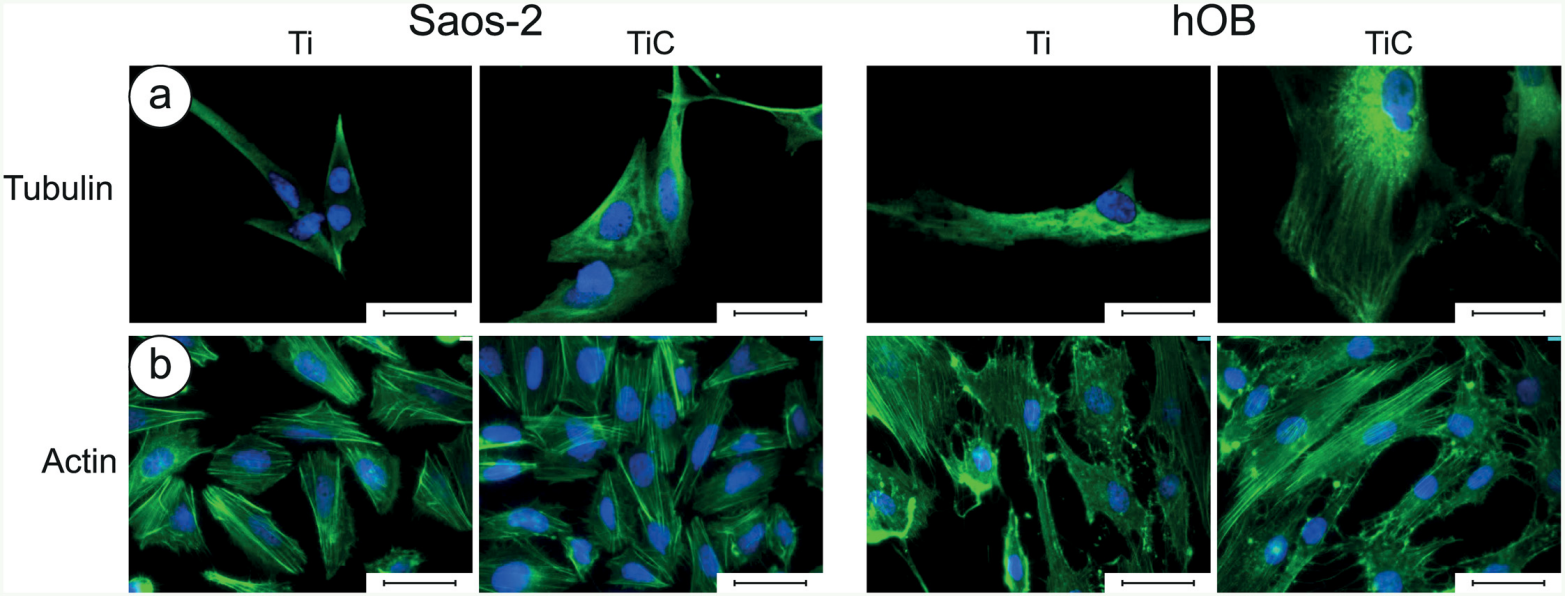
Immunofluorescence images of tubulin and actin in Saos-2 cells and in human primary osteoblasts. **Panel a:** The Saos-2 (left panels) and the hOB cells (right panels) were grown for 96 h on glass slides coated with 10.5 nm of titanium or the nanostructured TiC layer, treated with primary monoclonal antibodies against tubulin (tubulin mouse monoclonal antibody 10 μg/ml) and Alexa Fluor 568 goat anti-mouse secondary antibodies, diluted 1:500. **Panel b:** The cells were treated with Phalloidyn Alexa Fluor 488-conjugated diluted 1:10. In all images, the nuclei were stained with DAPI. The images were collected with a magnification of 63X, and the bar represents 100 μm.

The cells grown on the nanostructured TiC film also evidenced an improvement in the amount and in the quality of actin and tubulin (Fig 4). In particular, the analysis with anti-tubulin showed that the tubulin was better distributed and the cells were more spread and extended on the TiC substrates, compared to the cells seeded on Ti substrates (Fig 4a). The analysis with anti-actin showed that actin cytoskeleton was better defined in cells grown on TiC coated glasses, and the number and spreading of Saos-2 cells and particularly of hOB, were higher than of cells grown on Ti coated glasses (Fig 4b).

### Adhesion and blood clotting

**Fig 5:**
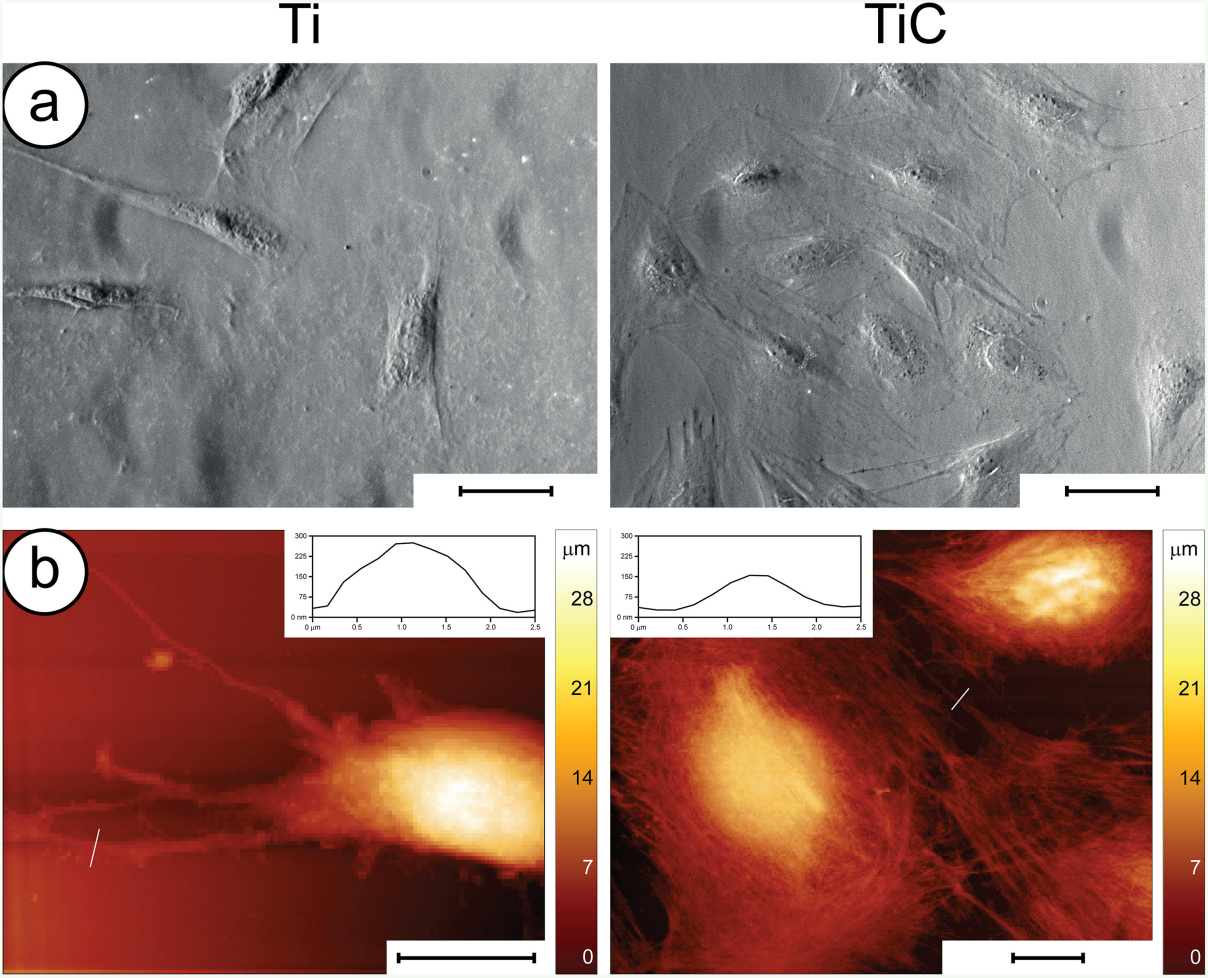
Adhesion of Saos2 cells on the nanostructured TiC surface. **Panel a**: Optical microscopy images of cells grown for 6h on Ti (left) and TiC coated (right) glass slides: the former exhibit cells with elongated form and fewer adhesion structures compared to those on TiC coated glass slides. The optical images were obtained using a 40x objective in phase contrast mode, the scale bar represents 100 μm. **Panel b:** AFM images of cells grown for 6h on Ti (left) and TiC coated titanium disks (right). The cells grown on the Ti coated substrates were rod-like with few large adhesion structures, highlighted in the inset. The cells grown on the TiC coated substrates appeared to have a stronger attachment with a larger amount of smaller filopodia and lamellipodia (shown in the inset) and with a more flattened form. The scale bar represents 10 μm.

At first, we performed conventional phase-contrast microscopy analyses, to evaluate the number and shape of the cells (Fig 5a). After 6 h of incubation, the Saos-2 cells grown on the TiC surface evidenced a round shape and appeared well attached to the substrate through many filopodia. On the other hand, the cells incubated on Ti coated glass slides resulted lower in number and different in shape, exhibiting an elongated form with fewer adhesion structures. We confirmed these findings by performing Atomic Force Microscopy (AFM) images of the cells grown on differently treated titanium disks (Fig 5b). The high-resolution images indicated that the cells grown on the nanostructured TiC surface resulted better spread and had a higher number of filopodia and lamellipodia when compared to those grown on the uncoated Ti surface.

**Fig 6:**
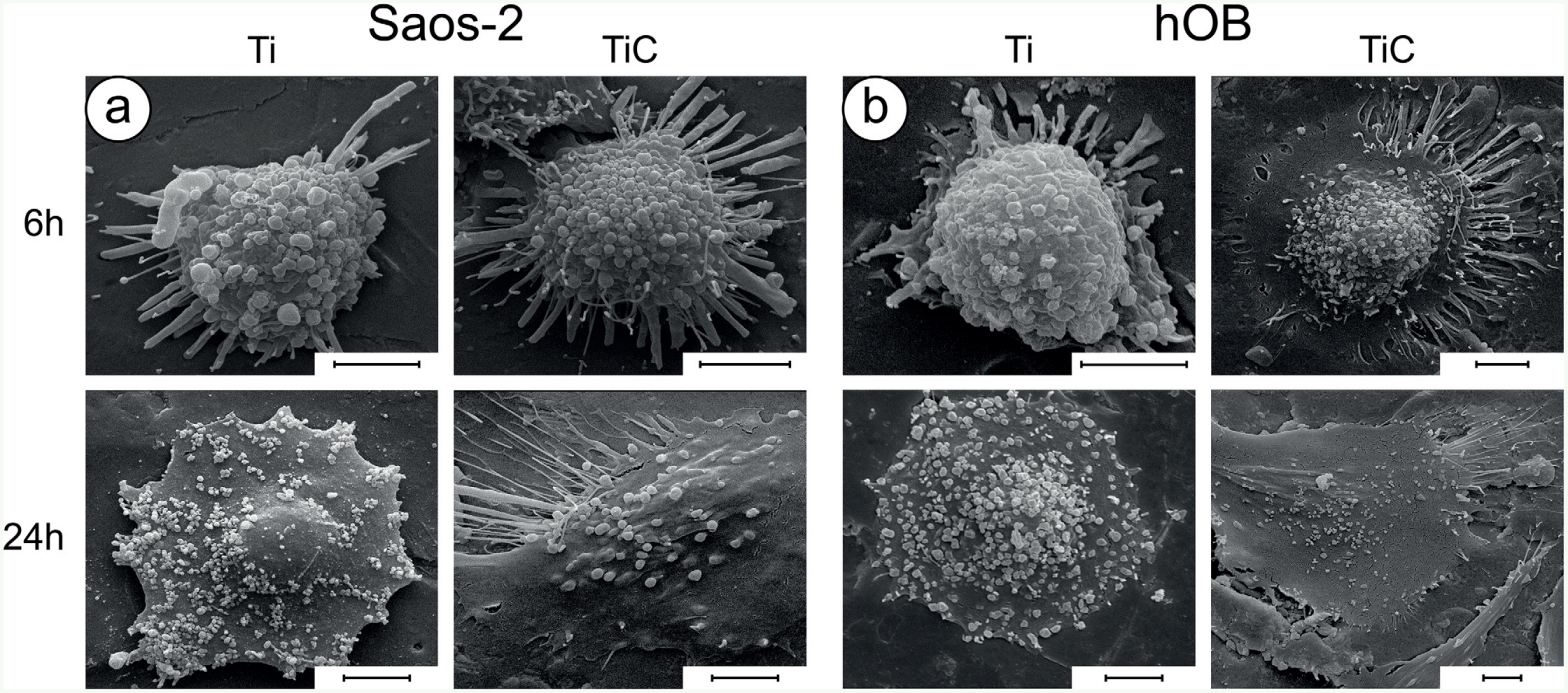
Investigation of cell morphology by SEM. **Panel a:** SEM micrographs showing the morphology of Saos-2 cells grown for 6h and 24h on uncoated (Ti) and TiC coated (TiC) titanium disks. **Panel b:** similar analysis on hOB cells. The images reveal that both types of cells are richer in philopodia and lamellipodia and better adhered to the substrate when grown either for 6 or 24 hrs on the TiC coated titanium disks compared to the uncoated. In each figure, the bars represent 5 μm.

To confirm these findings, we evaluated the adhesion fingerprint of the cells to the nanostructured TiC film using Scanning Electron Microscopy (SEM) analysing the Saos 2 cells (Fig 6a), and the hOB (Fig 6b). After 6 hours of incubation, all cells exhibited the rounded form, which is typical of the first phases of substrate adhesion. Yet, a comparison between the cells grown on Ti and those grown on TiC indicates that, even after just 6 hours, the latter possess a higher number of adhesive structures (filopodia and lamellipodia) compared with cells grown on Ti (Fig 6**, top panels**).

After a longer incubation (24 hours), the SEM images showed differences that were more marked (Fig 6**, bottom panels**). The presence of the TiC layer lead to flattening of the cells, which appeared well spread and with long cellular extensions. On the other hand, most of osteoblasts plated onto the Ti surface failed to express cellular extensions and maintained a shape that is reminiscent of the preliminary rounded form.

All these morphological results show that the nanostructured layer obtained by the IPPA deposition has a great effect on cell adhesion and spreading, greatly favouring the osteoblast colonization.

**Fig 7:**
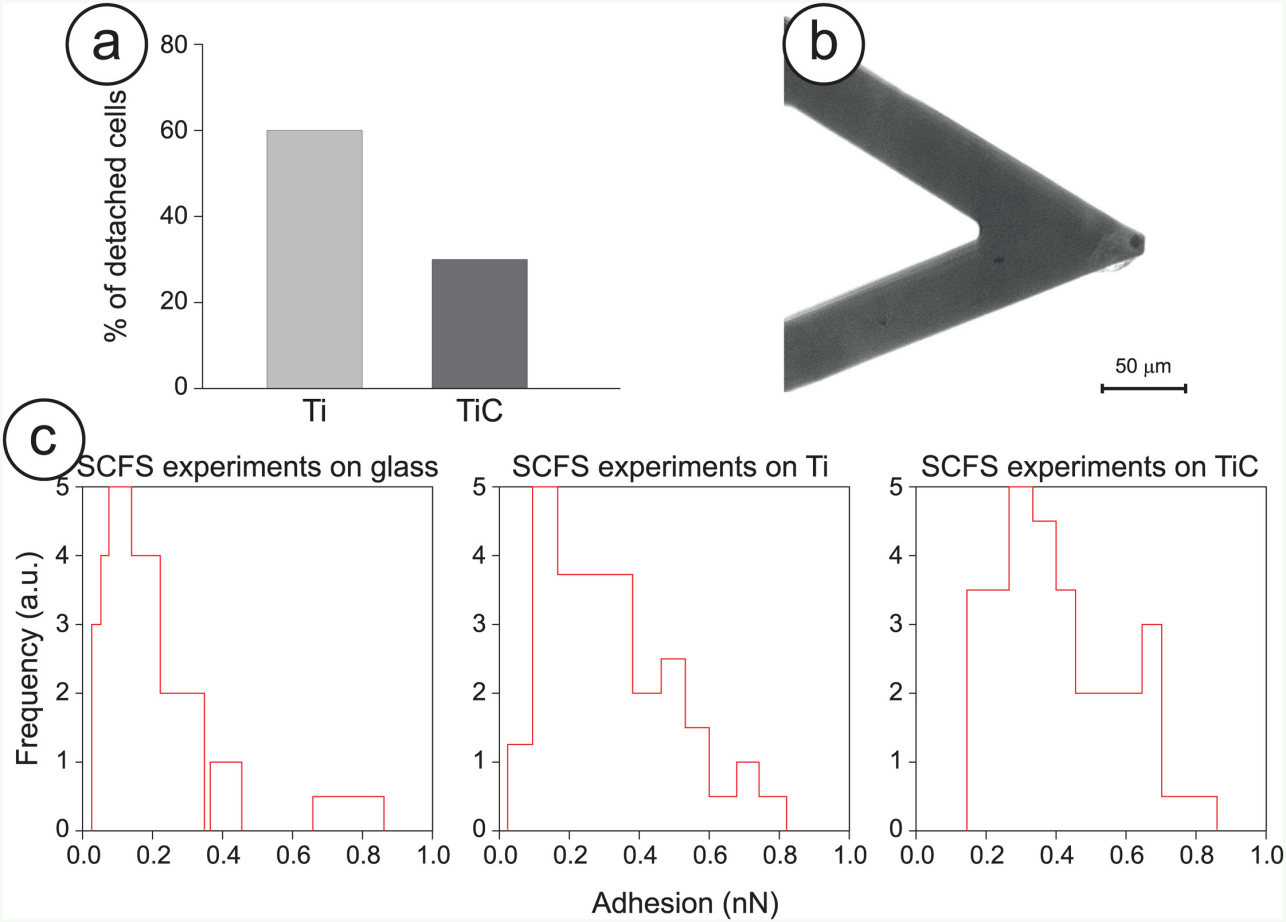
Adhesion strength of Saos-2 cells to the nanostructured TiC film. **Panel a:** Estimation of the detachment of Saos-2 cells grown on the titanium (Ti) and TiC coated titanium disks (TiC) for 6h upon exposure to the detaching buffer. **Panel b:** Optical image of a cantilever used for a SCFS experiment with an osteoblast cell firmly attached to the apical area of the sensor. **Panel c:** Comparison of the adhesion forces between the Saos-2 cells and bare, titanium and TiC treated glass substrates. For these experiments, the cells were placed in growing medium and placed in contact with the substrates using a maximum applied force of 6 nN for 20 seconds.

To better confirm these findings with more quantitative analyses, we evaluated the cell’s adhesion using both bulk and single-cell experimental procedures (Fig 7).

The bulk experiments involved exposing the adhered cells to a detaching buffer. These detaching experiments showed that, while about 60% of cells adhering to the untreated surface were removed, only 35% of cells adhering to the TiC coated surface were detached (Fig 7a). This demonstrates that the cells grown onto the nanostructured TiC surface exhibit a higher adhesive strength.

While this is a strong indication of the difference in cell adhesion between the untreated and the treated substrates, we performed a second assay with the innovative modality of the AFM called the single cell force spectroscopy (SCFS), to determine quantitatively the adhesion force of the cells on the differently treated substrates. Indeed, such a direct measurement is of paramount importance to confirm the biological properties of the TiC nanostructured layer. At first we fished one Saos2 cell from the culture medium with a chemically-modified AFM tip and we left it to adhere strongly to the cantilever. (Fig 7b) At this point the cell was placed in contact with the chosen substrate (glass, Ti or TiC) for a defined time and then pulled away, to measure quantitatively the adhesion between cell and surface. The resulting force curve measurements probed the cell-substrate interactions produced after three different times (5, 20 and 60 seconds) and allowed comparing the adhesion values that the cell’s interaction produced. The results of the adhesion experiments are summarized in Table 3 and the histograms of the SCFS experiments (20-sec) are depicted in Fig 7c. From each force curve, we extracted the value of the adhesion between the cells and the substrates. While no evident difference can be obtained from the 5-second experiments, in the 20-second and even more in the 60-second cases the adhesion values measured in the case of cell-TiC are much higher than the ones in the cell-Ti and cell-glass cases. These results were quite consistent when repeating the experiments using different cells. This indicates that the osteoblasts, in less than 20 seconds, are able to assay the chemical composition of the surface and start interacting with the substrate and that this interaction is larger in the case of the TiC nanostructured layer compared with the Ti film or the bare glass.

**Table 3:** Results of the SCFS experiments at different time scales.

Substrate	Adhesion after 5 seconds (pN)	Adhesion after 20 seconds (pN)	Adhesion after 60 seconds (pN)
**Glass**	346 +/- 239	165 +/- 85	368 +/- 205
**TiO**_**2**_	334 +/- 160	362 +/- 189	465 +/- 339
**TiC**	349 +/- 153	430 +/- 200	612 +/- 268

**Fig 8:**
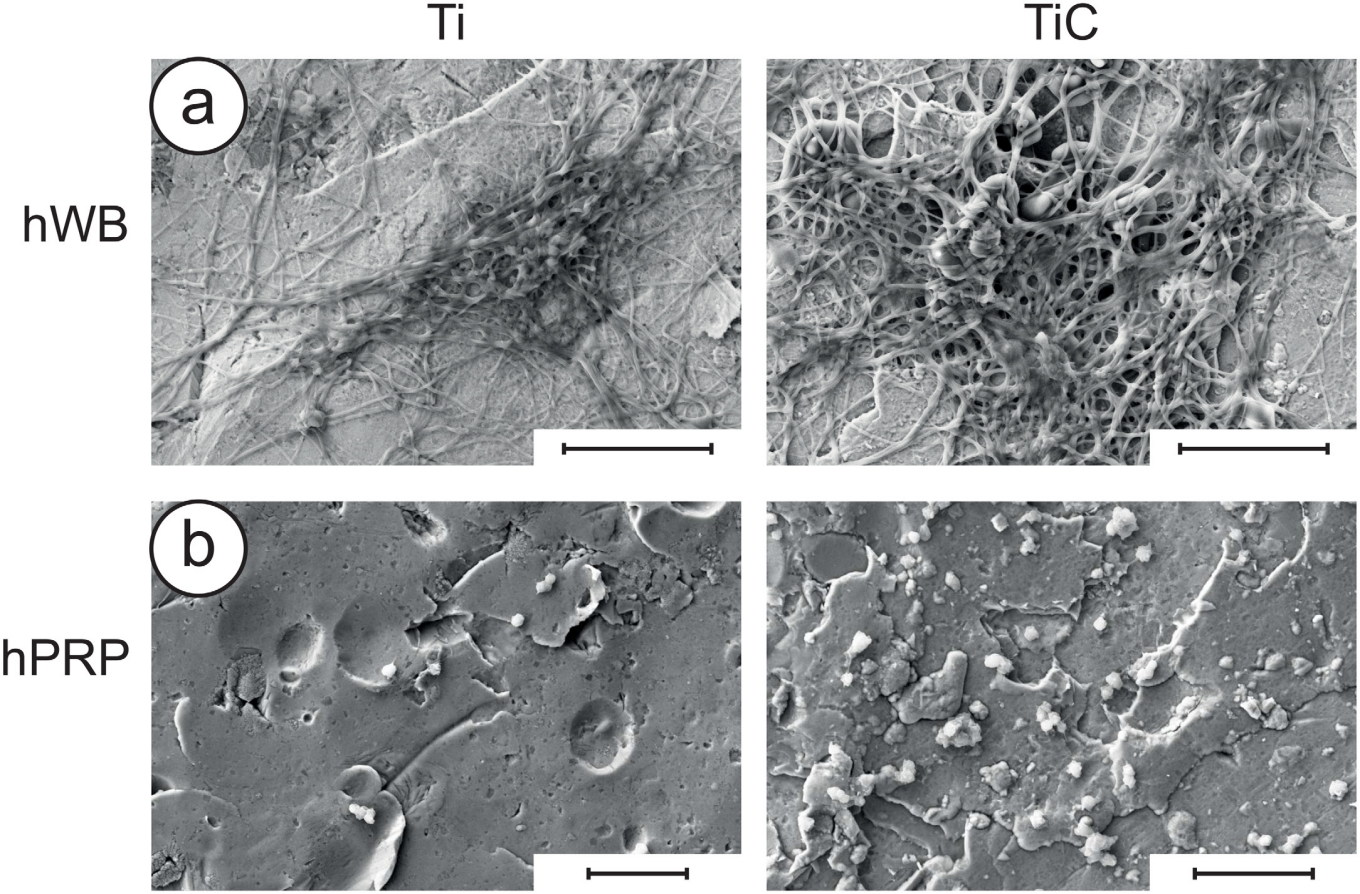
SEM analyses of micrographs of hWB and hPRP on differently treated substrates. **Panel a:** uncoated (left) and TiC coated titanium disks (right) treated with human Whole Blood (hWB) for 4 minutes. **Panel b:** uncoated (left) and TiC coated titanium disks (right) treated with human Platelet-Rich Plasma (hPRP) for 90 minutes. In both cases, the TiC coated titanium disks present a more efficient formation of blood clots and a higher number of adhering platelets, compared to the uncoated cases. In each figure, the bars represent 10 μm.

Blood clotting is the first interaction between an implant and the living tissue.[54] As consequence, the attractiveness of the nanostructured surface to whole blood, platelets and fibrin could play a very useful role in improving the speed of implant osseointegration. To verify this, we exposed the differently prepared substrates to human Whole Blood (hWB) and to Platelet-Rich Plasma (hPRP) observing with SEM the entity of their interactions. As reported in Fig 8, SEM micrographs show that titanium disks coated with the nanostructured TiC layer induce a more efficient blood clot formation and a higher number of platelets adhered to the coated titanium disks compared to the uncoated disks.

## Discussion

Overall, the data we have collected using different characterization techniques indicate that the nanostructure we have constructed, is composed of graphitic carbon, a highly biocompatible element, to which titanium oxides and titanium carbide are bound.[23] This layer combines the good biocompatibility of titanium oxides, which has been previously assessed by many investigations[8,55], and titanium carbide, which combines two biocompatible elements, such as carbon and titanium. All the titanium of the nanostructure is bound to oxygen or to carbon and both compounds are strongly bound to graphitic carbon in a very stable nanostructure. Remarkably, no free titanium is present, which may further react with oxygen.

The good biocompatibility of the TiC nanostructure is indicated by the absence of any toxicity, and by the production of ALP and mainly TGFβ1, which are higher in the test with osteoblasts grown on TiC coated than on uncoated titanium disks.

Cell adhesion is a very complex process, which comprises at least four major steps that precede proliferation: protein adsorption, cell-substrate contact, cell-substrate attachment, and cell spreading. Cell-substrate contact and cell-substrate attachment are strictly dependent on the chemical structure of the substrate [56,57] and on some physical parameters, such as the nano and micro roughness of the substrate, its wettability and its surface free energy.[21,22,38,39] Thus, a complete characterization of the substrate is the very first step in understanding the cell adhesion and proliferation.

The measurement of the contact angle between a liquid droplet and a surface is an extremely common analysis technique to determine hydrophilic/hydrophobic (wettability/non-wettability) properties. Specifically, if the contact angle value is less than 90°, the surface is hydrophilic (good wettability) or partially hydrophilic, whereas if the contact angle value is more than 90°, the surface is hydrophobic (lacking wettability).

To achieve a good adhesion to a substrate, firstly of the proteins of the extracellular fluid (i.e. fibrinogen) and then of the overall cells, the wettability of the substrate should have a value of about 70°. Furthermore, the adsorption appears to be improved on hydrophobic surfaces, compared to hydrophilic ones. The water contact angle of TiC coated samples showed a net increase in hydrophobicity of approximately 18%, compared to uncoated substrates that, with the decrease of the surface free energy, the absence of its polar fraction and the net increase of its basic fraction, favour the osteoblast adhesion we were able to measure through many further experiments.

Cells sense their local environment and respond to external chemical and physical cues creating a functional bidirectional cross talk.[58] In osteoblasts, this is initiated by integrins, a family of transmembrane receptors composed of α and β subunits that mediate the adhesion of cells to the extracellular matrix. Integrins are distributed on the surface of cells and are specifically stimulated by the appropriate chemistry of the environment. In particular, they interact with FAK (Focal Adhesion Kinase complex), which contain paxillin, talin and other proteins that are known to produce cytoskeletal changes in response to extracellular stimuli.[1,43–46] The β1 common subunit of integrins is coupled with a variety of different subunits: in osteoblasts, the most expressed is the dimer α3β1 [50], whose unique gene we have found overexpressed at early times in hOB grown on TiC coated glasses (3h, Fig 3a). Proteins of the FAK plaque, talin and paxillin, either of hOB or Saos2 cells, are both highly stimulated by the chemistry of the nanostructured TiC layer. (Fig 3b and 3c).

Integrins allow chemical signals to be transferred from the environment inside the cell, where these signals, amplified by cytoskeleton, arrive to the nucleus. This affects the gene expression by inducing cellular adhesion, spread and cellular migration, hence regulating the growth processes and cellular differentiation.[52,53] Immunofluorescence analysis showed that the inner nets of tubulin and actin are better defined either in hOB or in Saos-2 cells grown on TiC coated than on Ti coated glasses, demonstrating a better receiving and amplifying the chemical signal coming from the substrate.

We studied the ability of cells to interact with substrates also by optical microscopy, AFM and SEM, finding that the number of filopodia and lamellipodia produced either by hOB or by Saos-2 cells were higher when the cells were grown on the nanostructured TiC layer than on titanium layer both at the early times (6h) and later times (24h) (Figs 5 and 6). Furthermore, the cell adhesion experiments (detaching and SCFS experiments) demonstrated that the adhesion on TiC was higher than that found on Ti layer. These qualitative and quantitative experiments demonstrate that the adhesion forces developed by osteoblasts are higher when they grow on the nanostructured TiC layer.

Overall, the cells “sense” both the surface chemical structure and the micro and nano scale morphological properties of the surface through specific receptors for adhesive ligands. Their different behaviour when cultured on Ti or TiC surfaces confirms that the nanostructured layer has a beneficial role in the cell growth and differentiation.

## Conclusions

Titanium has numerous excellent features for implants and prostheses, such as the low density, low price, high robustness and high biocompatibility, but cannot be considered an ideal material. Indeed, its high reactivity to oxygen leads to the formation of a TiO_2_ layer, which is considered the source of the high biocompatibility of titanium prostheses. However in a not uncommon number of cases titanium implants may induce fibrinogenesis with a consequent implant loosening. As consequence, the prosthetic research has moved towards the study of coating procedures to improve the titanium surface, in its mechanical, morphological and chemical properties.

Overall, in this work, we present a vast amount of data that all point to the very same conclusion. A hard nanostructured TiC layer, produced by IPPA coating, bestows on titanium surfaces good mechanical, chemical and morphological properties, which produced excellent *in vitro* biological results. Such modified surfaces would improve the osseointegration process, stimulating osteoblast proliferation, adhesion and activity. The unique physical and mechanical properties of the layer will protect the titanium from the aggressive attacks of biological fluids and will increase the biocompatibility of titanium through surface stability as suggested by the stimulating effect on proliferation, adhesion and activity of osteoblasts. Finally, the Ion Plating Plasma Assisted deposition does not require over expensive equipment and the coating step can be introduced in any industrial process without changing the construction plans.

All these extremely important properties indicate that the IPPA coating with the TiC layer could become a useful step in the development of dental and bone implants. Protecting the titanium of the implant with a harder nanostructured TiC layer should reduce the risk of implant failure. In addition, the chemical composition of the layer, which increases adhesion, proliferation and differentiation of osteoblasts, is predictive of a better and faster osseointegration of the implant. Furthermore, the excellent properties and the absence of any toxicity will make this layer the ideal substrate for other stimulating agents, which will have an additive beneficial effect on the bone formation and cell proliferation.

## Supporting Information

S1 File Contains Substrate preparation, Measurement of the contact angle and determination of the surface free energy. **Fig A**, **XPS comparison between disks and glass substrates.** The XPS spectra were collected from: (A) Ti-coated glass slide; (B) Native passivating TiO_2_ layer on a Ti disk; (C) TiC-coated glass slide, and (D) TiC-coated titanium disk. The close correspondence between the coating layers produced on glass slides and on Ti disks ensures the chemical equivalence of the corresponding substrates.
(DOCX)
