# Supplementary material for: Improving Osteoblast Response In Vitro by a Nanostructured Thin Film with Titanium Carbide and Titanium Oxides Clustered around Graphitic Carbon
Source: PLoS One. 2016 Mar 31;11(3):e0152566. doi: 10.1371/journal.pone.0152566 (PMC4816526; doi:10.1371/journal.pone.0152566)
Supplement: S1 File — Fig A, XPS comparison between disks and glass substrates. The XPS spectra were collected from: (A) Ti-coated glass slide; (B) Native passivating TiO2 layer on a Ti disk; (C) TiC-coated glass slide, and (D) TiC-coated titanium disk. The close correspondence between the coating layers produced on glass slides and on Ti disks ensures the chemical equivalence of the corresponding substrates. (DOCX) [file pone.0152566.s001.docx]

**Supplementary Information**

**Improving osteoblast response *in vitro* by a nanostructured thin film with titanium carbide and titanium oxides clustered around graphitic carbon.**

Giovanni Longo^§^*, Caterina Alexandra Ioannidu^, Anna Scotto d’Abusco^, Fabiana Superti+, Carlo Misiano^&^, Robertino Zanoni^-^**,** Laura Politi^, Luca Mazzola^, Francesca Iosi+, Francesco Mura^-^, Roberto Scandurra^.

§ Istituto di Struttura della Materia, CNR, Via del Fosso del Cavaliere 100, 00133 Roma and Ecole Polytechnique Fédérale de Lausanne, SB IPSB LPMV, BSP 409 (Cubotron UNIL), R.te de la Sorge, CH-1015 Lausanne, Switzerland

^ Dipartimento di Scienze Biochimiche, Università di Roma ‘La Sapienza’, Piazzale Aldo Moro 5, 00185, Roma, Italy

+ Dipartimento di Tecnologie e Salute, Istituto Superiore di Sanità, Viale Regina Elena, 299, Roma, Italy

& Romana Film Sottili, Anzio, Roma

^-^ Dipartimento di Chimica, Università di Roma ‘La Sapienza’, Piazzale Aldo Moro 5, 00185, Roma, Italy

* Corresponding author. Giovanni Longo, longo@ism.cnr.it

**Substrate preparation**

To analyse the cells by fluorescence microscopy, to detect differences in cell growth on Titanium (Ti) and on the nanostructured Titanium carbide (TiC) film, we coated by IPPA (in optimal deposition conditions) some glass slides with very thin films (10.5 nm) of Ti or TiC. The layers allow the transmission of 25% of the light, which was sufficient to perform fluorescence microscopy analyses. We used XPS characterization to determine the chemical composition of the layers of titanium and TiC on the glass slides used for immunofluorescence experiments and to determine that they had the same composition with the corresponding titanium disks. This analysis is reported in **Figure A** and demonstrates that the corresponding substrates had identical composition.

Due to these results, we were able to perform immunofluorescence analyses directly on the treated glass slides and to compare these results with those obtained from cells grown on the disks.


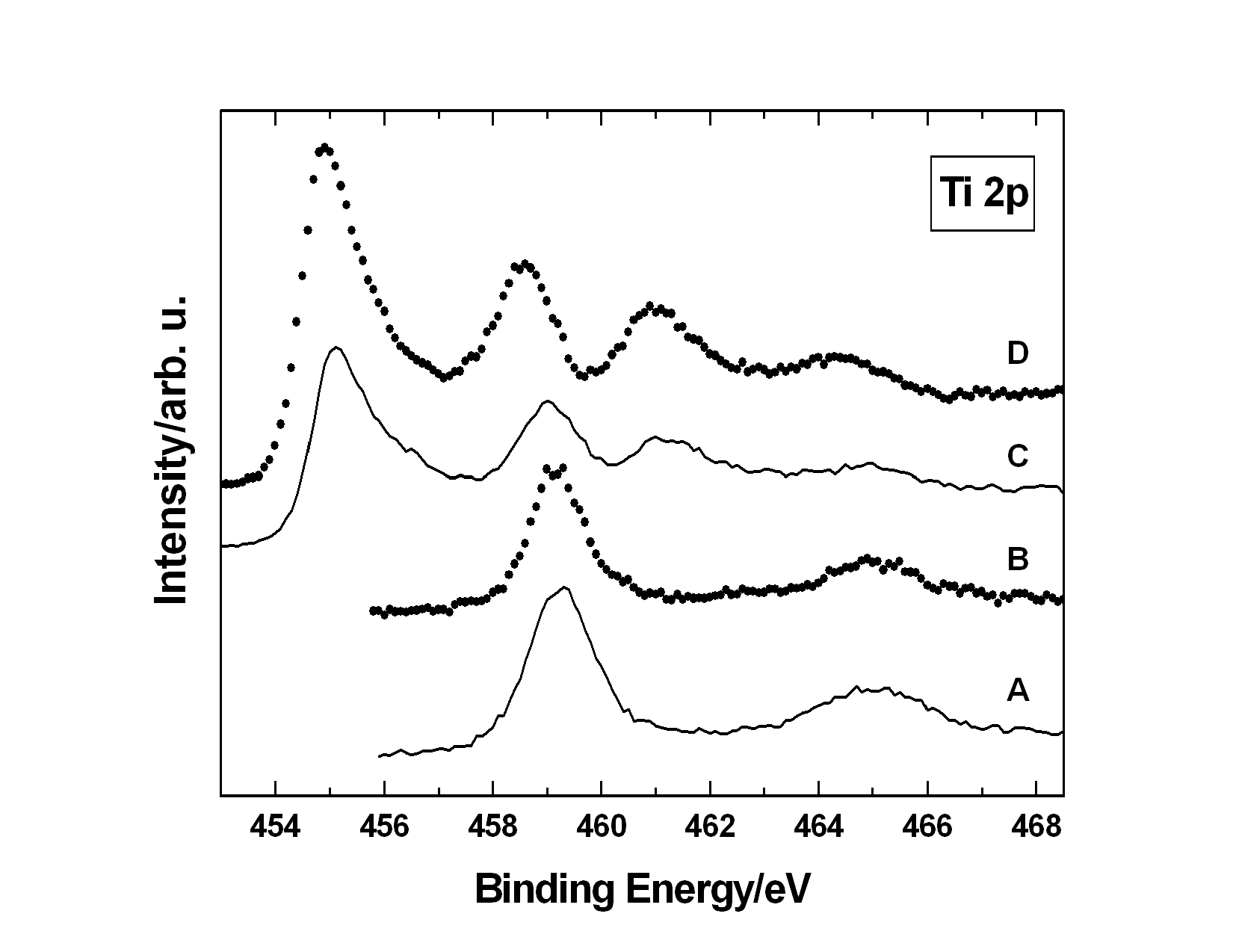


**Figure A**. **XPS comparison between disks and glass substrates.** The XPS spectra were collected from: (A) Ti-coated glass slide; (B) Native passivating TiO_2_ layer on a Ti disk; (C) TiC-coated glass slide, and (D) TiC-coated titanium disk. The close correspondence between the coating layers produced on glass slides and on Ti disks ensures the chemical equivalence of the corresponding substrates.

**Measurement of the contact angle and determination of the surface free energy**

The surface free energy was calculated according to the Van Oss – Chaudhury - Good method, which represents the best, and innovative method for calculating the surface free energy of the materials. Following this model, the wettability and the surface free energy of a surface follow the geometric mean relationship:


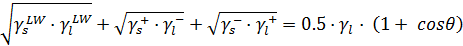

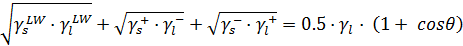
 (1)

where:

θ = contact angle between the liquid droplet and surface


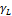

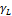
 = liquid total surface tension


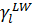

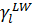
 = Lifshitz Van Der Waals component of liquid surface tension


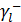

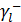
 = base fraction of liquid surface tension


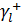

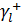
 = acid fraction of liquid surface tension

In our case, the last three parameters are unknown terms in this equation. The value of total surface free energy of the solid is obtained using the following equation:


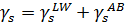

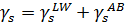


where


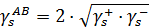

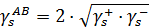


Substituting the data of the surface tension of each liquid and the relative contact angle (measured experimentally), we can solve the system of three equations in three unknowns, and determine the Lifshitz-Van der Waals component, the acid/base fractions and the total surface free energy of the different substrates.
